# Paediatric Palliative Care: Theory to Practice

**DOI:** 10.4103/0973-1075.76244

**Published:** 2011-01

**Authors:** Maryann Muckaden, Manjiri Dighe, PD Balaji, Sunil Dhiliwal, Prajakta Tilve, Sunita Jadhav, Savita Goswami

**Affiliations:** Palliative care services, Tata Memorial Centre, Mumbai; 1 Medical social worker department, Tata Memorial Centre, Mumbai; 2 Psychiatry unit tata Memorial Centre, Mumbai

**Keywords:** Paediatric palliative care, Cancer, Pain

## Abstract

Paediatric palliative care is a holistic approach aimed at addressing the complex issues related to the care of children and families facing chronic life limiting illnesses. The needs of children are unique and often quite different from those of adults receiving palliative care. This review article outlines some of the salient features of paediatric palliative care which are relevant to all professionals caring for children with life limiting illnesses in their practice.

“You matter because you are you and you matter to the last moment of your life. We will do all we can to help you, not only to die peacefully, but to *live until you die*”

*— Dame Cicely Saunders*

## INTRODUCTION

New advances in medicine have improved survival among children with any life-limiting illness. However, approximately 25% of these children would still die of their disease even after many years. When the hope of cure and prolonged survival dwindles, families and care givers may face tremendous stress. Care at this stage requires a holistic approach to the patients’ and families’ physical, emotional, and spiritual needs.[1] It should aim to achieve realistic goals. Such an approach improves clinical care and may also contribute to a sense of satisfaction and meaning that the physician can gain from the experience of caring for children at the end of life.

Palliative care for adults is now a recognized specialty worldwide; the same is not true for pediatric palliative care. Palliative care for children is in a much earlier stage of societal acceptance, and is only beginning to receive its rightful place in the spectrum of health care services. The strongly held belief that “children are not supposed to die” creates societal barriers to facing this reality.[2]

## WHAT IS PALLIATIVE CARE?

The term “palliative” is derived from the Latin word *pallium* meaning a cloak. Palliative care aims to cloak the patient’s symptoms and provide comfort even when treatments aimed at cure are no longer possible.

### Definition of pediatric palliative care

- Palliative care for children is the active total care of the child’s body, mind and spirit, and also involves giving support to the family.
- It begins when the illness is diagnosed, and continues regardless of whether or not a child receives treatment directed at the disease.
- Health providers must evaluate and alleviate a child’s physical, psychological and social distress.
- Effective palliative care requires a broad multidisciplinary approach that includes the family and makes use of available community resources; it can be successfully implemented even if resources are limited.
- It can be provided in tertiary care facilities, in community health centers and even in children’s homes. WHO 1998.[3]

The definition highlights several important points. Care is total, i.e. it addresses the physical, psychological, social and spiritual dimensions of suffering. The care extended to the patient is total. The concept of “total pain” was first introduced by Dame Cicely Saunders to encompass all four dimensions of pain.

Care extends to the child’s family. The family of a child with an advanced cancer faces various burdens. Palliative care providers recognize care giver stress and attempt to address it. Support to the family as a cohesive unit enhances care delivered to the patient. In India, financial strains, loss of work and social stigmas associated with cancer are the most common areas of stress for the family.

It is recommended that palliative care starts ideally at the time of diagnosis and extends all through the disease trajectory into bereavement support. Early intervention by the palliative care team will facilitate better “total” care from the outset. However, oncologists are often reluctant for early palliative care as the focus is on cure and the parents would loose hope.

The place of care can be a hospital, hospice or the child’s own home. The child and family’s preference for the place of care provision is respected as far as possible. This approach requires proper networking with local medical facilities, home care or access to local hospice.

The definition also stresses the importance of a multidisciplinary approach in best caring for a child with an advanced incurable illness.

### Which children benefit from palliative care

- Conditions where curative Rx feasible can fail, e.g. cancer.
- Conditions where, ultimately, death is inevitable – could be after years, e.g. cystic fibrosis.
- Progressive conditions – exclusively palliative, e.g. Baten’s disease.
- Irreversible but non-progressive disease, e.g. cerebral palsy.

### Pediatric palliative care in India

It is difficult to give exact figures, but it is estimated that 1/10 children with cancer, ½ children with cerebral palsy, 1/7 with human immunodeficiency virus, 1/10 with thalassemia, and 1/5 with neurodegenerative disorders will need pediatric palliative care. Another area of need is the neo natal realm, where children with severe birth asphyxia, complex congenital heart diseases and other disorders would need palliative care. Guestimates would put this figure at 3 lakh at any one time.

The place of care in India is a matter of grave concern. Most children with advancing disease hail from the villages, where trained pediatricians are few. Yet, the parents would like to return home and get the best possible medical assistance as there is the rest of the family to be cared for along with the sick child. Trying to identify innovative options to care for this stretched family takes all the skills available at the village level to be put to use. In practical terms, this involves the help of any doctor available to be trained telephonically by the pediatric palliative care team. The addition of a nurse is a boon. Most dressings and giving of medications, including reporting of side-effects, is carried out by the family. The extended family members help in caring for the needs of the child and siblings for psychological and social needs, including non-formal education.

### Ethics in pediatric palliative care

- The ethical principles that underlie advance care planning are the same as in other health care encounters – respect for autonomy, beneficence and non-maleficence.
- Any treatment considered should be in respect to the anticipated benefits and burdens, and should be undertaken only when the benefits proportionately outweigh the burdens. Herein lies the challenge – to predict benefits and burdens within the complex contexts of uncertainty and varying values.
- Care of child and family.

## PHYSICAL SYMPTOMS IN CHILDREN

Children with advanced cancers are likely to have multiple symptoms. Symptoms may not be adequately treated – lack of self-reporting by children, non-availability of proper assessment tools, unawareness or reluctance on the part of the doctors to actively seek symptoms, problems using certain drugs like strong opioids or availability and proper dosing.

Data regarding symptoms of children dying of cancer are limited. However, the more common symptoms experienced include pain, fatigue, nausea, vomiting, cough, anorexia and psychological symptoms.

A survey of children with cancer aged 10-18 years at Memorial Sloan Kettering, New York, showed a high symptom burden. The most common symptoms (prevalence >35%) were lack of energy, pain, drowsiness, nausea, cough, lack of appetite and psychological symptoms (feeling sad, feeling nervous, worrying, feeling irritable). Those that caused high distress in more than one-third of the patients were feelings of sadness, pain, nausea, lack of appetite and feeling irritable. The factors associated with a higher symptom load were – inpatient status, recent chemotherapy (in the last 4 months) and those patients with solid tumors vs. leukaemia, lymphoma or central nervous system malignancies.[4] Symptoms seen in children with advanced cancers are enlisted in Table 1.[4]

**Table 1:** Common symptoms at presentation at the Tata Memorial Centre

Fatigue	Dizziness
Pain	Numbness/tingling in hands/feet
Drowsiness	Sweating
Nausea	Lack of concentration
Cough	Diarrhea
Anorexia	Skin changes
Feeling sad, nervous, irritable, worrying	Dyspnea
Insomnia	Altered taste
Dry mouth	Oral ulcers
Hair loss	Dysphagia
Vomiting	Constipation
Weight loss	Problems with urination

When dealing with physical symptoms in a child with advanced disease, it may be important to remember that the goal at this stage is to maximize comfort and to improve the quality of life to the best possible, while weighing the possible side-effects and benefits of a given treatment. Investigations such as routine blood tests, recommended previously, have to be discontinued as the focus shifts to comfort care. Rather, the physician is called upon to be “low tech and high touch.”

### Pain

Pain is defined as “an unpleasant sensory and emotional experience with actual or potential tissue damage, or described in terms of such damage.”[5]

Multiple factors affect a child’s experience of pain. They include stable factors like age, gender, temperament and cultural background. The modifiable factors include cognitive, e.g. understanding of disease, perceived control; behavioural, e.g. staff responses, inappropriate drug prescribing or untreated side-effects, social isolation; emotional, e.g. depression, anxiety, fear of death.[6] Attempts to positively change these modifiable factors are likely to have a positive impact on pain control.

### Pathophysiology of pain in cancer

Pain may be classified as functional or pathological. Functional pains are visceral (e.g., colic) or somatic (e.g., cramps, tension, headaches). Pathological pain may be nociceptive (due to tissue injury or distortion) or neuropathic (due to nerve compression or injury).

Somatic pain is perceived as well localized and results from of activation of nociceptors in cutaneous and deep musculoskeletal tissues. Examples occur from bone metastasis, post-surgical incisional pain and pain accompanying myofascial or musculoskeletal inflammation.

Visceral pain results from infiltration, compression, distension or stretching of thoracic and abdominal viscera by the tumor (e.g., liver metastasis and pancreatic cancer). It is characterized as deep, squeezing and pressure and is poorly localized and often referred to cutaneous sites. Nausea, vomiting and sweating may accompany the pain when it is acute.[7]

Neuropathic pain occurs as a consequence of injury to the central or peripheral nervous system by compression or infiltration of peripheral nerves, nerve roots or the spinal cord due to tumor, surgical trauma, chemotherapy or radiation-induced injury. Common neuropathic pains include metastatic or radiation-induced brachial or lumbosacral plexopathies, epidural spinal cord and/or cauda equina compression, phantom limb pain, post-herpetic neuralgia and painful vincristine, cisplatin or paclitaxel neuropathy. Neuropathic pain is often severe and is characterized as a dull ache with “vice-like” quality, superimposed paroxysms of lancinating or electrical shock-like sensations are common. It may be in an area of absent sensation or allodynia.

Accurate assessment of pain in children is essential for correct management. Pain can be measured by a child’s behavioral response (e.g., facial expression) or physiologic response (e.g., tachycardia or self-report).

A framework to assess pain is called QUEST[8], i.e. Question the child, use a pain-rating scale, evaluate behavior and physiological changes, secure parental involvement, take the cause of pain into account, take action and evaluate results.

Reliable pain scales based on some form of self-report are available for assessment of children’s pain, and include the following:[9] [Table 2]

**Table 2:** Pain scales for children based on self report

Tool	Age group	Comment
Wong Baker Faces rating scale[10]	From 3 years	6 faces depicting smiling to neutral to total misery to denote pain severity
Poker chip tool[11]	4–8 years	4 poker chips placed in front of child – each is a piece of hurt
Eland color scale[12]	4–10 years	2 figures for the front and back of the child to depict location of pain and colors for severity
Numeric scale	From 9–10 years	10 cm line with 5 or 10 marks to depict no pain on extreme left to worst possible pain on extreme right
Verbal graphic rating scale[13]	9–15 years	Horizontal or vertical 10 cm line with descriptors starting from no pain on extreme left to worst possible pain on extreme right

Preverbal patients cannot communicate their pain in the standard, subjective manner. This inability to express and quantify their pain places preverbal children at risk for inconsistent identification of pain and inadequate pain relief.[14] Special scales have been developed for use in preverbal children (neonates, infants) and can be applied to children who are not able to communicate due to their illness or a comorbid condition. Most of these depend on observations of the child’s behavior or responses to existing pain. Some of these scales are: [Table 3]

**Table 3:** Pain scales for children unable to report their pain

Tool	Age	Comment
Children’s Hospital of Eastern Ontario Pain Scale (CHEOPS)[15]	1–5 years	Assess cry, facial expression, verbal, torso, touch, legs. Validated for 1–5 years. Post-op; good interobserver reliability
Nurses Assessment of Pain Inventory[16] (NAPI)	Newborn to 16 years	Assess verbal/vocal, body movement, facial, touching; adapted from CHEOPS, reliable
FLACC scale (F) Face; (L) Legs; (A) Activity; (C) Cry; (C) Consolability[14]	2 months to 7 years	Appropriate for preverbal children in pain from surgery, trauma, cancer or other disease processes

Management includes pharmacologic and non-pharmacologic measures.

**Figure 1:**
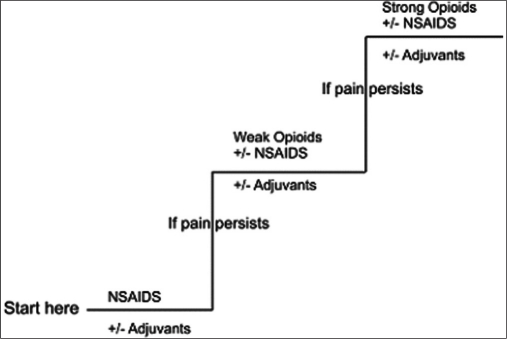
The WHO analgesic ladder for cancer pain

The guiding principles of analgesic administration are “by the ladder,” “by the clock,” and “by the mouth” and “by the child.”[2] In this context, the WHO ladder has revolutionized the effective management of pain. The WHO analgesic ladder is shown below in Figure 1.

#### Step 1

Paracetamol is the drug of choice (10-15 mg/kg. X Q4 hrly). It is safe, cheap and easy to administer. NSAIDS are another main group of drugs for treating mild to moderate pain. Ibuprofen, naproxen, diclofenac and acelophenac are commonly used NSAIDS. There are some who still use Cox-2 inhibitors due to its selective activity. Side-effects include hepatic, renal disturbances and bleeding due to platelet dysfunction

#### Step 2

Opioids are recommended for moderate to severe pain. They are classified as weak (e.g., codeine, tramadol) and strong (e.g., morphine, fentanyl). Opioids act through receptors that are found in several areas of the brain, particularly in the periaqueductal grey matter, and throughout the spinal cord. Supra-spinal systems have been described for some of these receptors, whereas some receptors modulate pain at the spinal level.[17] The specific effects of receptor activation are:

μ = Analgesia, respiratory depression, miosis, euphoria, reduced gastrointestinal motility

x = Analgesia, dysphoria, psychotomimetic effects, meiosis, respiratory depression

δ = Analgesia[2]

Codeine (methylmorphine) (0.5–1 mg/kg Q4 hrly) is a naturally occurring opium alkaloid used as an analgesic, antitussive and antidiarrheal agent Therapeutic effects are mediated via mu receptors. Codeine is one-tenth as potent as morphine and is usually combined with a non-opioid like paracetamol and/or NSAIDs.

Tramadol is one-fifth as potent as morphine orally. It acts via the opioid receptor and also blocks the pre-synaptic reuptake of serotonin and norepinephrine. It provides very quick analgesic action when given intramuscularly.

Dextrapropoxyphene has not been adequately tested in children and is thus not routinely recommended for use in children. It is, however, a useful drug with minimal side-effects and is being used as an off-label indication in centers with a lot of experience.

#### Step 3

Morphine by mouth is the global strong opioid for cancer pain (in India, the only one).(l8) In India, morphine is available as tablets, sustained release tablets, elixir and injections. The preferred route is oral, while other routes are subcutaneous, intravenous and per rectal. Oral morphine is half as potent as subcutaneous morphine and one-third as potent as intravenous morphine. Morphine undergoes pre-systemic metabolism in the liver and is excreted by the kidney; doses must be reduced in renal or liver dysfunction.

The starting dose of morphine is 0.15–0.3 mg/kg every 4 h. The duration of action is 3–4 h and hence every three to four hourly dosing is advised to maintain blood levels and prevent breakthrough pain. Morphine is recommended when pain is not adequately controlled with a weak opioid and a non-opioid. The dose can be titrated upwards till pain is relieved or intolerable side-effects develop.

The common side-effects of morphine are constipation, nausea, vomiting, xerostomia, urinary retention, sedation, hallucinations, myoclonus, itch and sweating.

Strong opioids like morphine do not cause clinically important respiratory depression in patients in pain.[18] Pain is a physiologic antagonist to the central depressant effects of morphine.[19]

It is important to prescribe a bulk-forming and stimulant laxative along with morphine to prevent constipation.

#### Adjuvants

Adjuvant analgesics are a miscellaneous group of drugs that relieve pain in specific circumstances.[20] These drugs are often used in the treatment of neuropathic pain. Some of the common adjuvants are listed in Table 4.

**Table 4:** Adjuvant analgesics

Drug	Dose	Action/group
Amitryptyline	0.2–0.5 mg/kg P.O. Titrate upward by 0.25 mg/kg every 2– 3 days	Neuropathic pain, antidepressant
Carbamazepine	Initial dosing: 10 mg/kg/day P.O. divided OD or BID. Maintenance: up to 20–30 mg/kg/day P.O. divided every 8 h	Neuropathic pain, especially shooting, stabbing pain, anticonvulsant
Gabapentin	5 mg/kg/day P.O. Titrate upward over 3–7 days. Maintenance: 15–50 mg/kg/day P.O. divided TID	Anticonvulsant; effective add on for neuropathic pain
Diazepam	0.025–0.2 mg/kg P.O. every 6 h	Acute anxiety, muscle spasm, sedativehypnotic
Prednisone, prednisolone, dexamethasone	Dexamethasone initial dosing: 0.2 mg/kg IV. Dose limit: 10 mg. Subsequent dose: 0.3 mg/kg/day IV divided every 6 h	Headache from increased intracranial pressure, spinal or nerve compression; widespread metastases

Non-drug measures for pain control

Non drug measures for pain control usually make pain more tolerable without affecting the intensity. Most of these are techniques for distraction and may include simple measures like holding a comforter, singing, going on an imaginary journey, watching TV, etc.[9]

### Fatigue

A study by Hinds *et al*.[21] involving children and adolescents revealed that the younger cohort characterized fatigue in terms of somatic complaints, like weakness with the hospital environment, frequent sleep interruptions, treatment complications, pain and low blood counts, as contributory and sleep and quiet, distracting activities, as beneficial factors. Adolescents described fatigue as an all-encompassing experience affecting their physical, mental and emotional state related to the amount of noise in the environment, negative emotions, boredom and insomnia. Helpful interventions were rest, leaving the treatment center, distraction, physical therapy and medical/pharmacologic relief of treatment-related complications.

- The exact pathophysiology of fatigue is not known. In adults, the possible factors are tumor-related lipolytic, proteolytic degradation factors produced by the neoplasm and direct endocrine abnormalities due to tumor infiltration. Host factors include host production of IL-6, TNF, etc. and co-existing conditions like anemia, uncontrolled symptoms, prolonged immobility with loss of muscle mass and reduced cardiac output, i.e. deconditioning, cachexia, hypoxia, etc.[22]

For treatment, the non-pharmacological measures are counselling, adapting activities of daily living and adequate rest or exercise. If deconditioning is a contributor to the fatigue, a change in medications perceived to be causing loss of energy and avoiding expending energy on unnecessary activities are to be practiced.[22]

Corticosteroids[23,24] and megestrol acetate[25] are known to be effective in the treatment of fatigue in adults, although the optimum effect is short lived. Correction of correctable causes like anemia, management of pain, depression, etc. where appropriate is essential.

### Anemia, cytopenias in pediatric palliative medicine

Hematological problems such as cytopenias are common in patients with advanced cancers. The causes are multiple and may co-exist in a given patient. Anemia may result from marrow failure (infiltration, radiation or chemotherapy), anemia of chronic disease, malnutrition, hemorrhage or chronic hemolysis. Timely blood transfusions can provide good relief of symptoms and improvement in the overall quality of life.

Indication for transfusion of packed cells is a hemoglobin value of <8 gm/dl. However, care must be taken to avoid fluid overload. Erythropoetin may be useful in the treatment of anemia, but cost is the prohibitive factor in a palliative setting.

Thrombocytopenia and leucopenia may also result from marrow failure. Symptomatic thrombocytopenia warrants platelet transfusion while hemostatic agents like tranexamic acid and ethamsylate may help.

### Nausea and vomiting

Nausea and vomiting are common symptoms in children receiving palliative care. Nausea and vomiting occur when the vomiting center in the brain is activated by any of the following: cerebral cortex (e.g., anxiety), vestibular apparatus, chemoreceptor trigger zone (CTZ), vagus nerve or by direct action on the vomiting center.

### Some of the causes are

#### Gastrointestinal

Gastric irritation, intestinal obstruction, constipation and hepatic distension.

#### Metabolic causes

Renal failure and hypercalcaemia.

#### CNS causes

Raised intracranial pressure.

#### Treatment of cancer

Medications (chemotherapy, opioids, etc.).

#### Psychological trigger

Anxiety and emotional distress.

#### Other causes

Pain and infection.

#### Situational triggers

Unpalatable food, etc.

Treatment includes correction of the correctable causes. The choice of anti-emetics depends on the underlying cause and the receptor being stimulated, e.g. the anti-emetic of choice for Morphine-related vomiting is Haloperidol. Where the cause is not immediately established, a universal anti-emetic is indicated. These include Metaclopromide and Domperidone in standard doses. The added advantage of these drugs is increased gastric emptying, which is usually delayed in these children. As second line of treatment, the antipsychotic drug, e.g. olanzepine, is very useful. Low-dose steroids may be added for refractory vomiting.

### Constipation

It is a common problem in palliative care and, if left untreated, can be quite troublesome to the patient.

Causes of constipation include: Tumor infiltrating or compressing the bowel, poor dietary and fluid intake, inactivity, metabolic e.g. hypercalcemia, drug induced e.g. opioids, anti-cholinergics.

Management of constipation includes “correcting the correctable,” as in most symptoms. These include dietary changes like more fiber, fluids, encouraging activity and addition of pharmacotherapy.

Laxatives may be frequently needed to treat constipation in palliative care. Laxatives are classified as bulk forming (e.g., isapghul), lubricants (e.g., liquid paraffin), surface-active agents (e.g., docusate), osmotic laxatives (e.g., lactulose, magnesium hydroxide, polyethylene glycol) and stimulant laxatives (e.g., bisacodyl, senna and sodium picosulphate). It is useful to use a combination of a softener and a stimulant laxative. Rectal measures may be required when oral laxatives are not effective. These include glycerine or bisacodyl suppositories followed by manual evacuation and a high-phosphate enema.

Patients who are on opioids must be given a bulk-forming and stimulant laxative prophylactically.

## PSYCHOLOGICAL ISSUES IN PEDIATRIC PALLIATIVE CARE

Children in palliative care have needs that are quite different from those of adults receiving similar care. Cancer-related deaths in children are less common than those in adults.[26]

Age-dependant cognitive abilities of children affect their perception of their illness, dying and control of the situation.[27] A young child up to 2 years of age does not have any concept of death, and treatment must be aimed at providing physical comfort. From 2 to 7 years, children may see death as a reversible process; it is important to minimize separation anxiety and to deal with guilt feelings. A child between 7 and 12 years of age is likely to understand the permanence of death, and may suffer from guilt, abandonment and fears of body mutilation. The adolescent faces a struggle between a need to be independent and possibly worsening physical symptoms and lack of control. Up to 1970, it was believed that children need not be informed about their illness and a closed protective approach was recommended. The work of Waechter[28] and Bluebond Langner[29] radically changed this view. They found that children with a fatal illness had extremely high levels of generalized anxiety even when the prognosis was not directly revealed to them. These children were also likely to depict loneliness, separation and death in their fantasy stories. Waechter noted a marked discrepancy between what the children actually knew and what their parents perceived they knew. Very few discussed death concerns with their parents. Denial by adults may not be entirely effective in preventing anxiety in children.

### Talking to children receiving palliative care

A study carried out at the Tata Memorial Hospital[30] highlighted the attitudes of parents toward information giving and support provided to the pediatric patient. Thirty-one parents were interviewed, of which 13 felt that the child was already aware without being told directly. Two were open to discussing the disease with the child, but felt that it would be difficult to talk about dying. A majority did not wish to discuss the disease or dying with the child, citing anticipated distress or young age as reasons for non-disclosure. In this study, parental anxiety and collusion were the major barriers to formal support or to open communication with the children.

A study by Kreicbergs *et al*,[31] in Sweden attempted to contact all parents who had lost a child to cancer between 1992 and 1997. Among 561 eligible parents, 449 answered a questionnaire, and 429 stated whether or not they had talked about death with their child. None of the 147 parents who talked with their child about death regretted it. In contrast, 69 of 258 parents (27%) who did not talk with their child about death regretted not having done so. Parents who sensed that their child was aware of his or her imminent death were more likely to regret not having talked about it.

Siblings were rarely told about the patient’s illness directly by the parents. Those who were informed were older children (siblings of two patients) who may be contributing to the families’ income or may accompany the patient to hospital in the absence of the parent.

Clearly, there is a difference in the psychological issues in pediatric palliative care in India and in the West. However, it is evident that children receiving palliative care have psychosocial needs that are not fully addressed. Research and training of staff dealing with such children is of paramount importance.

### Care of care givers

Families of children having advanced cancers face manifold stresses – emotional, financial, loss of job of parents, spending prolonged periods of time away from home for treatment and even social isolation in some cases. In a study from Israel, “Helping parents of the dying child – An Israeli experience,”[32] some concerns listed are:

- Cope with terminal illness.
- Cope with imminent death.
- Cope with social stigma.
- Cope with ill child.
- Prepare family for imminent death.
- Discuss fears.

In this setting, family care givers are at risk for burnout. Some form of respite care in the form of day care facilities or facilities for short stay may be helpful in preventing such burnout. However, such facilities are often lacking in our country.

Within the constraints of pediatric palliative care services that presently exist in our country, providing support groups for children and families in a non-threatening environment may be useful. Such groups can be formed by enlisting the help of families themselves along with the support of trained staff and voluntary organizations.

## BEREAVEMENT SUPPORT

The death of a child is regarded by Western societies as one of the most painful bereavements.[33] Deaths carrying a stigma, such as deaths from AIDS or suicide, or deaths for which the bereaved carries some responsibility, also bring a higher risk of poor outcome.[33] Most palliative care services in the West include bereavement support as an essential part of the services they provide. This is because various studies in the West have shown the positive impact of such services on the bereaved family. Bereavement support is provided by various methods like telephonic follow-up, home visits, individual visits, condolence cards, memorial letters, self-help programmes, family therapy, group therapy, etc. Telephonic calls are a very common form of bereavement follow-up. A study by Kaunone *et al*, suggested that the bereaved needed the telephonic call. This study has suggested the need for bereavement interventions.[34] One of the studies on intervention like family-focused grief therapy was focused for those at risk families to prevent the complication of the bereavement and abnormal grief. It focused on communication of thoughts and feelings, cohesion, handling of conflicts and enhancing the functioning of the family. The study showed that there is decreased distress levels and improvement in protecting the bereaved from pathological grief; thus, concluding that psychotherapy for high-risk families has positive outcomes.[35] In Individual therapy, there are various ways where individuals can be supported. These include telephonic follow-ups, individual meetings, one to one counselling or self-help groups. Self-help groups can be very useful in adjusting to new situations, enhancing coping skills by sharing the experiences and feelings. It becomes easy when the person sees another person facing a similar situation and also easy to see how one is coping. A study by Vachon *et al*, involving women who had lost their husbands showed that the participant’s adaption to the loss was improved after widows were paired with another widow who was trained in counselling and who managed her grief well. Results showed that the participant’s adaption to the loss was improved compared with the control.

An unpublished study conducted by the palliative care team from the Tata Memorial Centre showed that our cultural and spiritual beliefs help in coping with loss much better as compared with those from the West, because of which the incidence of abnormal grieving is less. In another study exploring the various coping mechanisms used by Indians in an urban setting, support through the bereavement phase comes from extended family and neighbours. Specific mourning rituals, the need to get on with living, earning a livelihood and the presence of other children in the family are factors that may help in the bereavement process.

## ADVOCACY AND NETWORKING IN PEDIATRIC PALLIATIVE CARE

Because India is a vast country with children suffering from life-limiting conditions all over the country in remote villages, the help of trained pediatricians will be essential to develop effective pediatric palliative care close to the child’s home. This will require a lot of advocacy for them to get involved. Networking with professionals of different specialties will help children in remote villages get the help they and their families need to die with peace and dignity at home.

Pediatric palliative care is its inception stage in India. Wider application and acceptance of this specialty is essential for the best possible care of children with advanced cancers.
